# Present situation and development prospects of the diagnosis and treatment of rotator cuff tears

**DOI:** 10.3389/fsurg.2023.857821

**Published:** 2023-06-27

**Authors:** Tianjun Zhou, Chang Han, Xisheng Weng

**Affiliations:** ^1^ Department of Orthopedic, Peking Union Medical College Hospital, Chinese Academy of Medical Sciences & Peking Union Medical College, Beijing, China; ^2^ State Key Laboratory of Complex Severe and Rare Diseases, Peking Union Medical College Hospital, Beijing, China

**Keywords:** rotator cuff tear, musculoskeletal ultrasound, arthroscopy, glucocorticoid, minimally invasive surgery

## Abstract

Rotator cuff tears are an important cause of shoulder pain and are caused by degeneration or trauma of the shoulder tendon at the anatomical neck of the humeral head. The understanding and research of rotator cuff tears have a history of hundreds of years, and their etiology, diagnosis, and treatment have a complete system, but some detailed rules of diagnosis and treatment still have room for development. This research paper briefly introduces the diagnosis and treatment of rotator cuff tears. The current situation and its valuable research direction are described.

## 1. Introduction

The rotator cuff muscle group is composed of the subscapular, teres minor, supraspinatus, and infraspinatus muscles. Its tendon forms a sleeve-like structure at the anatomical neck of the humeral head. The most important function of the rotator cuff is to ensure the stability of the shoulder joint (1). The estimated prevalence of shoulder pain in the population is approximately 16%–34% (2, 3), and rotator cuff problems are one of the main causes of shoulder pain. According to the statistics conducted by the U.S. Department of Health and Human Services in 2004, the number of visits caused by shoulder pain was approximately 4.5 million, and the number of rotator cuff surgeries was up to 20,000 (4). Therefore, public health problems caused by rotator cuff tears are common and important. The diagnosis and treatment of rotator cuff tears is a focus of clinical medicine, especially orthopedics.

## 2. Pathogenesis

At present, etiological research on chronic rotator cuff tears can be summarized into two kinds: degenerative changes or exogenous impacts.

As early as 1934, Codman proposed that the main cause of rotator cuff tears was tendon degeneration (5). Rothman performed rotator cuff angiography in 1965 and found that the tendons of the supraspinatus and infraspinatus muscles had clear ischemic areas, and ischemia led to the degeneration of the tendons, resulting in a decrease in rotator cuff strength, injury, and tear (6). In 1972, Neer questioned the theory of degenerative change (7). He suggested that 95% of rotator cuff tears were caused by acromion impact. The movement of the shoulder joint causes the rotator cuff to be slightly impacted by the acromion and coracoacromial arch, resulting in congestion and edema of the rotator cuff, which may eventually lead to tendon rupture (8).

Therefore, the incidence rate of rotator cuff tears increases with age (9). At the same time, for occupations requiring a wide range of upper limb activities, especially throwing athletes, the probability of rotator cuff tears in youth is significantly higher than that in the general population (10). In addition, a study involving 180 patients with rotator cuff injuries (11), showed that diabetes, obesity, hyperlipidemia, hypertension, and smoking habits were risk factors for rotator cuff injury.

## 3. Clinical manifestation

### 3.1. Symptom

The most common complaints of patients with rotator cuff tears are pain and fatigue. The pain site is mainly around the deltoid muscle, and the pain is usually aggravated when the upper limb moves widely or the shoulder is compressed (12). However, in subsequent studies, the correlation between pain and rotator cuff tears was questioned. First, in Itoi's study (13), the main cause of shoulder pain was synovial inflammation caused by rotator cuff tears, not tendon injury. Another series of studies pointed out that the severity of rotator cuff tears seems to have little relationship with the severity of pain, and more than half of rotator cuff tears have no symptoms (14). Compared with complete tears and partial tears, the traditional understanding is that complete tears will bring more severe pain (15), but some studies also reported that the tear range will not affect the pain index (16) or that the pain caused by partial tears is higher than that caused by complete tears (17). Therefore, the significance of shoulder pain in the diagnosis of rotator cuff tears remains to be further discussed.

Because rotator cuff tears are essentially tendon injuries, shoulder fatigue is also one of the common complaints of patients with rotator cuff tears. For complete or large-area tears, fatigue is a particularly important symptom (18). It should be noted that when distinguishing between rotator cuff tears and fatigue caused by pain, such as synovitis and osteoarthritis, local anesthetics are often used to check muscle strength after injection. Fatigue caused by rotator cuff tears will not be relieved by local anesthesia.

### 3.2. Physical examination

There are various physical examination methods for the shoulder, and the effect of those methods varies greatly. Therefore, there is no perfect examination method to accurately diagnose rotator cuff tears. In 2005, Park et al. conducted a study involving 552 patients (19) and using arthroscopy as the diagnostic standard, they verified eight common shoulder joint examination methods, of which three were selected as the diagnostic examination methods of rotator cuff tears: active painful arc test, drop arm test, and weakness in external rotation. The combination of the three methods can achieve high diagnostic efficiency. If the three methods were positive, the final diagnosis was a rotator cuff tear, whose likelihood ratio was 15.6. However, if the three methods were negative the final diagnosis was no rotator cuff tear, whose likelihood ratio was 0.16, meaning that the scheme can achieve quite a good diagnosis or exclusion effect. Therefore, these three inspection methods are the most commonly used in a large number of physical examination methods. Another study pointed out that the Hornblower sign, that is, shoulder joint external rotation pain when resisting applied force, can be used alone for the examination of rotator cuff tears, and the sensitivity can reach 70% (20).

However, at present, research on shoulder physical examination is still insufficient. There is not enough high-quality evidence in evidence-based medicine to determine one or a group of examination methods that can effectively diagnose rotator cuff tears (21). Therefore, relevant research needs to be further promoted, and the clinical diagnosis needs to be combined with the medical history, imaging, and other methods.

### 3.3. Imaging examination

#### 3.3.1. X-ray

At present, x-ray is not routinely used in the clinical diagnosis of rotator cuff tears, especially chronic rotator cuff tears. However, an x-ray can detect the relative position changes of the humeral head and acromion, which is helpful to diagnose complete tears. An image of a noticeable humeral displacement can confirm a large-scale tear and, at this time, the rotator cuff tendon can be fully involved (22). Although x-rays do not satisfactorily meet the current imaging needs, with the progress at the medical level, the diagnostic ability of rotator cuff injury still has a certain value in rural clinics lacking medical conditions.

#### 3.3.2. Musculoskeletal ultrasound (MSK US)

Compared with other imaging techniques, ultrasonography has inherent advantages: no radiation, low cost, and convenient bedside examination (23). Some clinicians who have received ultrasound training can use MSK US for rapid and accurate initial evaluations (24). In a study that included 331 cases of rotator cuff surgery (25), taking the surgical results as the gold standard, MSK had a sensitivity of 79% and a specificity of 94%. In addition, many diagnostic studies have shown that MSK is a very effective diagnostic method (26–28). The manifestations of rotator cuff tears in MSK US include but are not limited to tendon thickening with hypoechoic, tendon calcification, tendon fiber rupture, and steatosis (29).

#### 3.3.3. MRI

MRI is very sensitive to soft tissue injury, so it can be used to diagnose rotator cuff tears. It is effective in the diagnosis of complete tears, but many studies suggested that the sensitivity of MRI to partial tears is not enough to meet diagnostic needs (30, 31). In patients with rotator cuff tears, MRI findings include but are not limited to tendon discontinuity, fluid signal generation, acromial osteophyte generation, and rotator cuff atrophy (32). A further diagnostic method is MR arthrography. When the MRI image is normal but there is a clinical suspicion of a rotator cuff tear, MRI can be used to observe whether the contrast agent goes deep into the acromion space or subdeltoid space after the intra-articular injection of the gadolinium contrast agent.

From the perspective of comprehensive imaging examination, the combined use of multiple imaging can more accurately diagnose most patients with rotator cuff tears, but there are still deficiencies compared with other common orthopedic diseases, especially fractional rotator cuff tears. It is suggested that comprehensive imaging examination, medical history, and physical examination should be combined to help in the diagnosis (33).

## 4. Treatment

The treatment of rotator cuff tears depends on many factors, such as tear range, tear time, shoulder joint mobility, and patient age (34). For such a surgical disease, the focus of research is whether surgery can be beneficial. In most current studies, even in patients with complete tears, the benefit of surgical treatment is not clearly higher than that of conservative treatment (35, 36). In an observational study of 4,542 patients with complete tear (37), functional improvement was not related to the treatment regimen after 1 year of intervention. However, in another long-term follow-up study (38), compared with the scores of function and symptoms after 10 years of intervention, surgical intervention was significantly better than conservative treatment. The explanation of the abovementioned results may be the instability of the area with the tear or tendon steatosis (39), but this was not confirmed by rigorous pathological tests in the current study. At present, the only patient population strongly recommended for surgical treatment is young patients with acute complete tears with severe symptoms and limited function.

### 4.1. Conservative treatment

The main purpose of conservative treatment after rotator cuff tears is to reduce symptoms and improve function (40). However, in patients with chronic tears, due to uncertain surgical indications, the effective rate of definite improvement within 1 year ranges from 33% to 92% (41). The key point of conservative treatment is rehabilitation exercise for the tissues near the shoulder joint. In the early stage, the joint capsule was stretched to improve the joint range of motion. In the middle stage, self-weight or light resistance exercise is used to restore muscle strength, and in the later stage, all-around shoulder training is carried out to improve joint coordination (42). Rehabilitation exercises should gradually change from passive exercise to active exercise. To restore function, rehabilitation exercises should be carried out as soon as possible. Theoretically, the treatment plan should be adjusted according to the degree of tear, muscle atrophy, and patient needs, but some studies have pointed out that adjusting the treatment plan for partial tear and complete tear has little effect on functional rehabilitation (40). In the clinical trial conducted by Zhang et al. (43), it was confirmed that active and passive shoulder joint activities can effectively promote tendon healing and improve shoulder joint function. Sheard et al. (44) added muscle strength training and nerve coordination training based on joint function training to achieve a better rehabilitation effect. Mahure et al. (45) used transcutaneous electrical nerve stimulation (TENS) for patients with rotator cuff injury in rehabilitation, and the results showed that inflammation and pain were effectively controlled, which could, to some extent, replace traditional analgesic methods. In the clinical research conducted by Bennell et al. (46), patients were completely allowed to practice after learning the exercise program by themselves, and the rehabilitation effect was not significantly different from that of the auxiliary treatment of the rehabilitator.

In addition to rehabilitation training, rotator cuff injury can benefit from glucocorticoid injection: to a certain extent, it can reduce local inflammatory reactions and relieve pain. Early injection of glucocorticoids after acute tear can quickly and effectively relieve pain and improve function, but it has no therapeutic effect on rotator cuff injury itself (47). Studies have shown that glucocorticoid injection therapy has better short-term efficacy and safety than placebo, oral non-steroidal anti-inflammatory drugs, and physical therapy in pain relief, activity, and function improvement (48).

In most cases, after the broken ends of tendons are repaired and sutured by surgery, the broken ends can be repaired and healed well within 6 weeks. However, several studies have shown that tendon rupture caused by glucocorticoids has poor repair and healing between the broken ends after surgical repair, and it easily ruptures again in the long term (49, 50). This shows that glucocorticoids can not only lead to spontaneous rupture of the tendon but also reduce the repair function of the tendon itself, and its mechanism is not clear yet (51). Spontaneous rupture of tendons caused by glucocorticoids has always been a very difficult problem in the field of sports medicine. In the 2019 clinical guidelines (52), moderate evidence supports that a single injection of corticosteroids combined with local anesthetics can improve the pain and function of patients with shoulder pain in the short term. At the same time, it is necessary to pay attention to the negative effects of multiple injections of corticosteroids on rotator cuff tissue.

In addition, massage, acupuncture, local injection of nitroglycerin, and other methods will also be used to treat rotator cuff tears in the clinic (53). Because the therapeutic effect has not been confirmed by sufficient data, it will not be discussed here. In addition, it should be noted that in some patients with rotator cuff tears treated conservatively, the severity of tears will gradually increase. Pain or fatigue will increase (54), and clinically, it will aggravate muscle injury and increase the difficulty of treatment. Therefore, it is recommended that all patients treated conservatively should have shoulder physical examination and imaging examination every 6–12 months (55).

### 4.2. Surgery treatment

At present, there are three common surgical methods: open surgery, small incision surgery, and arthroscopic surgery. In open surgery, the deltoid muscle needs to be completely open and then sutured after the rotator cuff tendon is exposed as much as possible. After rotator cuff injury, surgical injury of the deltoid muscle will cause the affected shoulder to completely lose function for a period of time (56), and the risk of infection is high. Therefore, compared with other less invasive surgical methods, open surgery has rarely been used.

Small incision rotator cuff repair was systematically applied by Levy in 1990 (57). Compared with open surgery, it has fewer intraoperative deltoid injuries, a good rotator cuff reconstruction effect, an obvious improvement in early postoperative quality of life, and a significantly shortened hospital stay (58). In a long-term follow-up study (59), patient satisfaction and treatment effect were basically the same as with arthroscopic treatment.

Arthroscopic repair of rotator cuff tears is the most widely used technique. Arthroscopy has natural advantages, including a small incision, a clear surgical field, small muscle injury, and a low infection rate (60). With the gradual upgrading of arthroscopic instruments and the improvement of arthroscopic operation technology, its clinical efficacy is also improving. At present, there are many methods of arthroscopic repair. The use of suture anchors, patches, tendon nails, and other auxiliary materials can effectively improve the repair effect (61).

As the trauma caused by arthroscopy is small and the postoperative inflammatory reaction is weak, in the research conducted by Kang (62) and Cho (63), the postoperative pain of arthroscopic surgery was far less than that of open surgery, which is significant for postoperative rehabilitation training. In addition, Nazari conducted a meta-analysis (64) on the range of motion of the shoulder joint after surgery, and the results showed that both arthroscopy and open surgery could achieve good improvement. As for shoulder joint function, the clinical research conducted by Walton et al. (65) showed that the recovery speed of shoulder joint function after arthroscopic surgery is much faster than that of open surgery, which is closely related to the small trauma brought about by arthroscopy. As the main method of rotator cuff tear surgery, multifaceted research on arthroscopic repair surgery is still in progress and has broad development prospects.

For massive rotator cuff injuries that cannot be repaired directly by tendons, tendon transfer is a therapeutic option that can effectively improve the function of the shoulder joint. In 1988, Gerber et al. (66) first applied this surgical method to separate the latissimus dorsi tendon from the humerus and fix it at the greater tubercle of the humerus to replace the broken supraspinatus and infraspinatus muscles. In 2012, a meta-analysis (67) showed that latissimus dorsi transfer could effectively improve shoulder joint range of motion and muscle strength, and the Constant shoulder joint score increased from 56 to 80 on average. For the anterior rotator cuff injury, such as subscapularis, the operation of pectoralis major muscle transfer can be selected. In a prospective follow-up study conducted by Moroder (68), the patients receiving pectoralis major muscle transfer surgery had significant recovery of shoulder joint range of motion within 10 years, especially the internal rotation movement. Pain relief and shoulder joint function scores were improved as well. In addition to the two most commonly used methods, trapezius, teres major, deltoid, and pectoralis minor can be used for tendon transfer, which can be adjusted according to the specific location of the injury. The main limitation of tendon transfer in rotator cuff injury is that the transferred tendon is prone to rupture or partial rupture, resulting in poor surgical effect, local hematoma, frozen shoulder, and other complications (69).

In addition to tendon transfer, various surgical methods can also be used for massive irreparable rotator cuff tears so that patients receive satisfactory treatment. Gartsman (70) reported a 79% satisfaction rate after open debridement and subacromial decompression in patients with massive irreparable rotator cuff tears. This kind of operation will decrease the pain of patients and improve the range of motion of shoulder joints and the ability to do daily tasks but will cause muscle strength decline (71), so it is more suitable for elderly patients with low functional requirements. Because of tendon retraction or excessive tissue tension, partial rotator cuff repair can also be the treatment option for huge rotator cuff injuries (72). Moser et al. (73) compared the surgical effects of complete repair and partial repair, and found that in patients with massive rotator cuff tears, a complete repair would bring a greater range of motion of the shoulder joint, while there was no statistical difference between pain and functional scores, which are more critical for the evaluation of surgical effects. In addition, there is the option of using auto-graft fascia lata as a patch to restore the superior capsule to its physiological state (74). Minhata et al. (75) followed up on the patients who underwent the superior capsule reconstruction for 2 years and found that the function of the shoulder joint was significantly improved, and there was no report of complications such as postoperative adhesion.

The subacromial spacer is another effective method for treating massive rotator cuff injuries. It uses arthroscopy to place biodegradable implants between the acromion and the humeral head, reducing friction between the rotator cuff injury site and the bone structure (76). While relieving pain, it also increases the abduction force arm of the shoulder joint, restoring the shoulder joint function. In the clinical cohort of Piekaar et al. (77), during a 3-year follow-up period, all 44 patients showed good pain relief and functional improvement. In a systematic review conducted in 2019 (78), the adverse reaction rate of this treatment method was only 3%, and the Constant-Murley shoulder joint function score was still satisfactory 2–3 years after surgery. However, in the study by Ruiz Ibán et al. (79), the patient satisfaction rate for this treatment method was only 40%, with 31.3% of patients experiencing increased pain 10 months after surgery and requiring a second surgery. Therefore, the effectiveness and safety of subacromial spacers are currently unclear.

Finally, reverse total shoulder arthroplasty (rTSA) can also be used as one of the treatment options for massive irreparable rotator cuff tears. Although rTSA can improve the pain and functional limitation of massive rotator cuff tears (80), its application is limited by the high rate of complications and revisions (81), so it is not recommended to use this surgical method in young patients (82).

During postoperative recovery, there are reports of the use of bioactive substances to promote the biological healing of rotator cuff tendons, such as platelet-rich plasma, mesenchymal stem cells, and cytokines gel. In cytological studies and animal experiments, PRP achieved beneficial results (83). In a meta-analysis that included 1,045 clinical data (84), PRP reduced the failure rate of rotator cuff injury surgery by more than 25%. However, in some clinical randomized trials, the effects of PRP on pain, function, and postoperative recovery were not statistically significant (85, 86). Gulotta et al. (87) demonstrated the repair ability of MSCs using the mouse rotator cuff injury model. In the clinical research conducted by Hernigou et al. (88), MSCs not only improved the degree of postoperative biological healing but also reduced the incidence of secondary laceration during the 10-year follow-up. The high expression of some cytokines, especially TGF-β3, in fetal trauma is believed to be related to scarless repair (89). In the mouse rotator cuff injury model of Han et al. (90), the gel containing TGF-β3 achieved a beneficial adjuvant therapeutic effect after injection. However, relevant research has not yet entered the clinical stage.

## 5. Summary

Rotator cuff tears have been recorded for a long time and are common in the population, especially asymptomatic rotator cuff tears. The etiology is clear, and the clinical diagnosis of complete tears and wide-range tears is relatively simple, but the identification of single tendon tears is still challenging and needs to be combined with several physical examinations and imaging to obtain a comprehensive diagnosis. The treatment is mainly conservative to maintain and restore function and surgical treatment with arthroscopic repair is the main treatment. Although the surgical indications are not completely clear, there is a great difference between young patients with definite symptoms and elderly patients with chronic tears. At present, the directions that are considered to be of more research value include efficient clinical diagnosis methods, more methods of conservative treatment, research related to arthroscopic surgery, and assisted recovery with platelet-rich plasma or mesenchymal stem cells.
